# AraDiv: a dataset of functional traits and leaf hyperspectral reflectance of *Arabidopsis thaliana*

**DOI:** 10.1038/s41597-023-02189-w

**Published:** 2023-05-24

**Authors:** Maria Stefania Przybylska, Cyrille Violle, Denis Vile, J. F. Scheepens, Benoit Lacombe, Xavier Le Roux, Lisa Perrier, Lou Sales-Mabily, Mariette Laumond, Mariona Vinyeta, Pierre Moulin, Gregory Beurier, Lauriane Rouan, Denis Cornet, François Vasseur

**Affiliations:** 1 CEFE, Univ Montpellier, CNRS, EPHE, IRD, Montpellier, France; 2 LEPSE, Univ Montpellier, INRAE, Institut Agro Montpellier, Montpellier, France; 3 Plant Evolutionary Ecology, Institute of Ecology, Evolution and Diversity, Faculty of Biological Sciences, Goethe University Frankfurt, Max-von-Laue-Str. 13, 60438 Frankfurt am Main, Germany; 4 IPSIM, Univ Montpellier, CNRS, INRAE, Institut Agro Montpellier, Montpellier, France; 5 Microbial Ecology Centre, UMR 1418 INRAE, UMR 5557 CNRS, INRAE, CNRS, University Lyon 1, University of Lyon, VetAgroSup, Villeurbanne, France; 6 CIRAD, UMR AGAP Institut, F-34398 Montpellier, France; 7 UMR AGAP Institut, Univ Montpellier, CIRAD, INRAE, Institut Agro, F-34398 Montpellier, France

**Keywords:** Ecophysiology, Evolutionary ecology, Evolutionary genetics, Plant evolution, Plant ecology

## Abstract

Data from functional trait databases have been increasingly used to address questions related to plant diversity and trait-environment relationships. However, such databases provide intraspecific data that combine individual records obtained from distinct populations at different sites and, hence, environmental conditions. This prevents distinguishing sources of variation (*e.g*., genetic-based variation *vs*. phenotypic plasticity), a necessary condition to test for adaptive processes and other determinants of plant phenotypic diversity. Consequently, individual traits measured under common growing conditions and encompassing within-species variation across the occupied geographic range have the potential to leverage trait databases with valuable data for functional and evolutionary ecology. Here, we recorded 16 functional traits and leaf hyperspectral reflectance (NIRS) data for 721 widely distributed *Arabidopsis thaliana* natural accessions grown in a common garden experiment. These data records, together with meteorological variables obtained during the experiment, were assembled to create the AraDiv dataset. AraDiv is a comprehensive dataset of *A. thaliana*’s intraspecific variability that can be explored to address questions at the interface of genetics and ecology.

## Background & Summary

Functional ecology has long used comparative approaches across species and environments to identify general patterns of trait variation linked to individual performance (functional traits^1^). In this quest for general trait patterns, the development of large trait databases, such as the TRY Plant Trait Database^2^, has become an important ally. Notably, it has allowed the demonstration that plant worldwide phenotypic variation and ecological strategies are better explained by multidimensional spaces summarized by both vegetative (*e.g*., specific leaf area) and reproductive (*e.g*., seed mass) traits^3^. Some lines of evidence, such as trait-environment relationships^4^, have suggested that the trait syndromes that underlie these multidimensional spaces are driven by adaptive processes. However, such an adaptive hypothesis still needs to be directly tested. For that, intraspecific trait data are valuable, but still lacking in functional trait databases^5^. Though these databases do present multiple records for a given species, such records come from distinct populations at different sites and, hence, environmental conditions. As a consequence, confounding effects prevent the distinction of sources of trait variation, which is a necessary condition for the study of evolutionary processes.

In order to deal with confounding effects and produce robust intraspecific trait data, experimental approaches like common gardening have been developed^6^. Common garden experiments consist of growing individuals from different populations under common conditions to assess the genetic basis of traits while controlling for the effects of phenotypic plasticity^6,7^. Another condition for a robust estimation of intraspecific variation is to assess a representative range of variation through the study of contrasted populations in terms of environmental conditions of origin^5,8^. Accordingly, more functional trait data both measured under common conditions and covering populations across the species’ geographic range are needed. Though logistically challenging, measuring intraspecific variation as described above within networks of standardized common garden experiments in different geographic zones can be a compelling perspective^9^.

Additional initiatives to make intraspecific trait data even more powerful to address questions at the interface of ecology and evolution is to link them with whole-genome data. Notably, this has been done with natural populations of *Arabidopsis thaliana*, *Medicago truncatula*, and *Populus trichocarpa*, allowing the study of genomic signatures of adaptation^10^. To promote more studies of adaptation, compilations of phenotypic data have been developed for genotypes whose sequencing had been previously made available to the scientific community (*e.g*., AraPheno^11^, PHENOPSIS DB^12^). Though such initiatives are valuable, they still largely lack key traits that functional ecology studies endorse as those capturing plant form and function (such as the vegetative and reproductive traits previously mentioned). Therefore, intraspecific information in these databases is likewise limited in its applicability to test hypotheses about the evolutionary determinants of plant trait syndromes and ecological strategies.

Leveraging trait databases with intraspecific trait data faces the major challenge of large-scale cross-population phenotyping. In recent years, the development of high throughput phenotyping methods, such as near-infrared spectroscopy (NIRS), has made phenotyping faster and easier, promoting intraspecific trait analyses^13^. Notably, commonly investigated functional traits underlying resource economics have been shown to be well predicted from the near-infrared reflectance value of leaves in *A. thaliana*^14^. Moreover, reflectance spectra have been applied to predict various chemical traits according to specific interests (*e.g*., secondary metabolites^15^ and phytohormones^16^) and they have also started to be used directly as phenotypic dimensions^13^. This last option can be interesting when lacking empirical data for trait prediction and when there is a special interest in analyzing as many phenotypic dimensions as possible, even those whose biological meaning remains unknown^13^. For instance, phenotype-blind approaches in plant biology for breeding selection have recently benefited from hyperspectral data^17,18^.

**Fig. 1:**
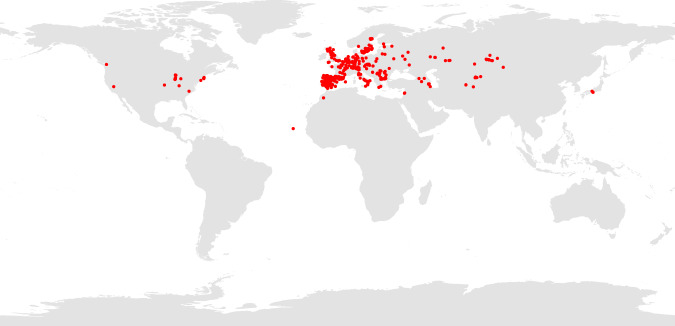
Origin of the natural accessions (red dots) assessed in this study.

**Fig. 2:**
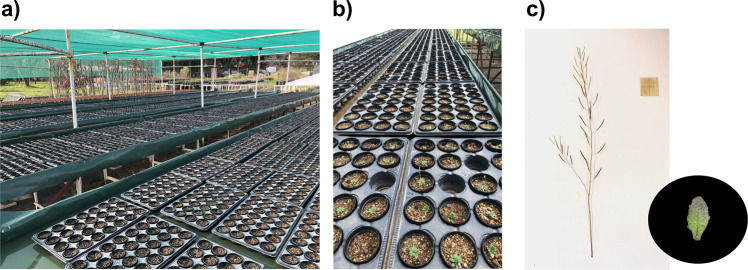
Photographs of the common garden experiment (**a,b**) and example of images used for trait analyses (**c**).

Here, we present the AraDiv dataset, which provides phenotypic and leaf hyperspectral reflectance (NIRS) data for 721 widely distributed *A. thaliana* accessions (Fig. 1). Phenotypic data include vegetative, phenological, and reproductive functional traits, constituting a comprehensive dataset of *A. thaliana*’s intraspecific phenotypic diversity. Phenotyped accessions were grown in a common garden (Fig. 2a,b), and meteorological data recorded during the experiment were also included in AraDiv. By phenotypically analyzing a subset of the accessions that are genetically and geographically (GPS coordinates) described in the 1001 Genomes Project^19^ dataset (http://1001genomes.org/), we intend to complement information about *A. thaliana*’s intraspecific diversity with a large amount of functional trait data obtained from plants grown under common conditions. This ultimately aims at fostering studies at the interface of genetics and ecology that link phenotypic, genetic, and environmental data to understand plant adaptation.

## Methods

### Experimental setup

A completely randomized common garden experiment was conducted between February and July 2021 under shade cloth in the experimental field of Centre d’Ecologie Fonctionnelle et Evolutive (CEFE), Montpellier, France (Fig. 2a,b). Pots of 0.08 L were filled with a sterilized soil mixture composed of 50% river sand, 37.5% calcareous clay soil from the experimental field at CEFE, and 12.5% blond peat moss. Seeds used in the experiment were distributed by NASC (https://arabidopsis.info/). Before sowing, we selected a total of 730 accessions from the 1001 Genomes Project^19^ (http://1001genomes.org/), based on Exposito-Alonso’s *et al*. list of accessions for maximizing the geographic and genetic coverage of *A. thaliana*^20^. At the beginning of February, all 730 accessions were sown in triplicates for harvest at flowering, and one replicate per accession was attributed to one of three blocks. At the end of February, a subset of 529 accessions were sown in three other replicates for harvest at fruit maturation, and one replicate per accession was attributed to one of three other blocks. Supplementary Table 1 shows the list of the 713 accessions harvested at flowering and 505 accessions harvested at fruit maturation, in a total of 721 accessions that germinated and reached the phenological stage of harvest. Experimental blocks were placed on growing benches adapted with a subirrigation system, and plants were irrigated three times per week until the end of the experiment.

### Phenotypic data

The recorded phenotypic data correspond to 16 functional traits (Table 1). Eight vegetative traits recorded at the beginning of flowering (first flower at anthesis), two phenological traits related to flowering, and six reproductive traits recorded at fruit maturation (first mature fruit). Common phenological stages were chosen for harvest since *A. thaliana*’s functional traits are known to vary across ontogeny^21^. The traits that were measured are likely to inform us about plant strategies both at the leaf and the whole-plant level, analogous to cross-species observations on plant strategies^3^.

**Table 1:** Measured traits and their units.

Trait type	Trait name	Trait unit
Vegetative	Leaf area	mm^2^
	Leaf area per leaf dry mass	mm^2^/mg
	Leaf dry mass	mg
	Leaf dry mass per leaf fresh mass	mg/g
	Leaf fresh mass	g
	Leaf nitrogen content per leaf dry mass	%
	Leaf thickness	μm
	Whole plant dry mass	mg
Phenological	Days to flowering	days
	Growing degree days to flowering	°C days
Reproductive	Fertility related trait	—
	Fruit length	cm
	Fruit number	—
	Inflorescence length	cm
	Secondary branch number	—
	Seed dry mass	mg

**Fig. 3:**
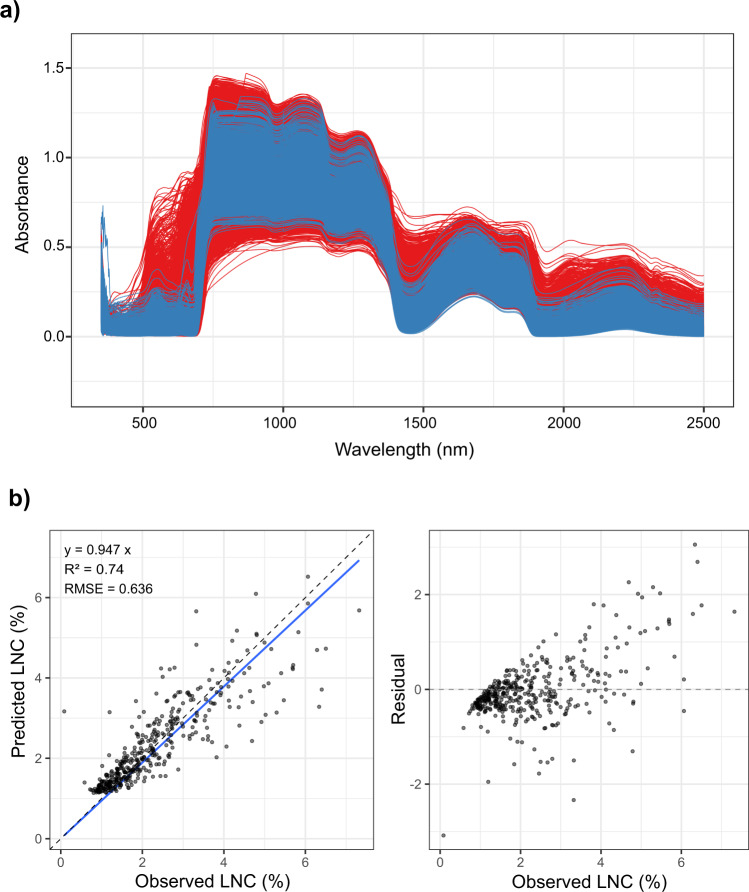
(**a**) Near infrared spectra used for model development (blue) and produced in this study (red). (**b**) Leaf nitrogen content per leaf dry mass (LNC) prediction model performances (left plot) and residuals (right plot). The solid blue line represents the fitted linear regression curve (with its formula in the top left corner), while the dashed lines show the equality line. R^2^: coefficient of determination (*N* = 403); RMSE: root mean square error.

After pots had been subirrigated for at least two hours, to promote leaf and whole-plant rehydration^22,23^, we recorded leaf fresh mass and leaf area for one leaf per plant (leaf selection followed methods described in Pérez-Harguindeguy *et al*.^23^). For that, the selected leaf was cut and right after weighed and photographed. One-sided projected leaf area (Fig. 2c) was measured through image analysis using ImageJ 1.53k software^24^. We recorded leaf and whole-plant dry mass after drying the samples at 60 °C for at least 72 hours. Leaf nitrogen content per leaf dry mass (LNC) was predicted using near infrared spectroscopy (NIRS, see below) and the predictive model developed by Vasseur *et al*.^14^ (Fig. 3a,b). To check NIRS prediction accuracy (see Technical validation), LNC was measured on a subset of 403 dried leaf samples (weighing 0.1 to 1 mg) using an elemental analyzer (Vario-PYROcube, Elementar, UK). Only predicted LNC values were incorporated into the phenotypic data record of the AraDiv dataset. Leaf thickness was estimated through the inverse of the product of specific leaf area (leaf area per leaf dry mass, m^2^ kg^−1^) and leaf dry matter content (leaf dry mass per leaf fresh mass, mg g^−1^)^25^. We calculated days to flowering from the sowing day until the first flower at anthesis. Days to flowering were also expressed in growing degree days (GDD), that is, the daily cumulation of Celsius degrees (°C) from sowing until the flowering date. Daily GDD was computed as ${\rm{GDD}}=\frac{Tmax+Tmin}{2}-{\rm{Tb}}$, where T_max_ is maximum temperature, T_min_ is minimum temperature, and T_b_ is base temperature, which was considered as 4 °C, the temperature usually used for cold acclimation and vernalization of *A. thaliana*^26,27^. T_max_ and T_min_ were obtained from meteorological data collected in the experimental field at CEFE (see below). For the measurement of reproductive traits, we cut inflorescence stems at their base and photographed them (Fig. 2c). Fruit (*i.e*., silique) length and number, inflorescence length, and secondary branch number were subsequently recorded using a macro, adapted from Vasseur *et al*.^28^, in ImageJ 1.53k software^24^. Fruit length was measured as the mean length of three randomly selected mature fruits per plant. Fertility was estimated through the product of mean fruit length and fruit number^29,30^. For measuring seed dry mass, inflorescence stems were dried at ambient temperature after harvest, and around 30 seeds per plant were weighed for an estimation of individual seed mass.

### NIRS spectral data

NIRS screening was performed using the ASD LabSpec 4 Hi-Res Analytical Instrument (Malvern Panalytical Ltd., UK). We screened rehydrated leaves right after they had been cut and leaf fresh mass and leaf area had been measured. The leaf spectrum of light reflectance was recorded for the spectral region 350–2500 nm (Fig. 3a), using a contact probe with a 10 mm spot size. The leaf midrib could not be avoided during NIRS screening due to a generally small leaf size. Only leaves that were big enough to be completely covered by the NIRS probe were screened. Acquired spectra are provided as a data record (see Data records).

### Meteorological data

Meteorological data were recorded using a weather station Davis Vantage Pro2 (Davis Instruments Corporation, USA) installed in the experimental field at CEFE. Ten meteorological variables (Table 2) were recorded every 30 minutes to derive daily means for the period of the experiment, *i.e*. from February to July 2021.

**Table 2:** Recorded meteorological variables and their units.

Meteorological variable	Unit
Barometric pressure	hPa
Evapotranspiration	mm
Maximum solar radiation	W/m^2^
Maximum temperature	°C
Maximum wind speed	km/h
Mean temperature	°C
Minimum temperature	°C
Rainfall	mm
Solar radiation	W/m^2^
Wind speed	km/h

## Data Records

The AraDiv dataset is composed of three data records, which have been deposited in the data.InDoRES repository (10.48579/PRO/SW1OQD)^31^ and are described below.

### Phenotypic data record

Phenotypic data are organized following the ecological trait‐data standard proposed by Schneider *et al*.^32^. Accordingly, data were recorded in a tidy format with the following 12 columns:

1. scientificName: a taxonomic reference to *A. thaliana* that follows the standard developed by the National Inventory of the Natural Heritage (https://inpn.mnhn.fr/accueil/index?lg=en).
2. X1001g_ID: accession number from the 1001 Genomes Project^19^ dataset (http://1001genomes.org/).
3. verbatimOccurrenceID: user‐specific identifier defining individual plants. Letters “P” and “A” mark individuals harvested at flowering and at fruit maturation, respectively.
4. verbatimBlockID: user‐specific identifier defining experimental blocks. Letters “P” and “A” mark blocks dedicated to plants harvested at flowering and at fruit maturation, respectively.
5. DateSowing: sowing date.
6. DateHarvest: harvest date.
7. HerbivoryIndex: qualitative herbivory index, ranging from zero to five, defined for the technical validation of reproductive traits (see Technical validation).
8. traitName: trait names according to plant ontologies, namely the Thesaurus of Plant characteristics (TOP^33^), the Plant Trait Ontology (TO^34^), and the Crop Ontology (CO^34,35^).
9. traitValue: trait values.
10. traitUnit: trait units.
11. traitID: Uniform Resource Identifier (URI) for applied trait ontology.
12. taxonID: URI for taxon identification.

### NIRS spectral data record

NIRS spectral data file comprises 2,152 columns. The first column is equivalent to the “verbatimOccurrenceID” column previously described, and remaining columns comprise leaf hyperspectral reflectance values in spectral regions from 350 to 2500 nm.

### Meteorological data record

Meteorological data were recorded in a tidy format with the following four columns:

1. Date: date from 1^st^ February to 31^st^ July 2021.
2. varName: meteorological variable names.
3. varValue: meteorological variable values.
4. varUnit: meteorological variable units.

## Technical Validation

A completely randomized design was adopted in the common garden experiment to minimize confounding effects related to the placement of the pots.

### Phenotypic data

**Fig. 4:**
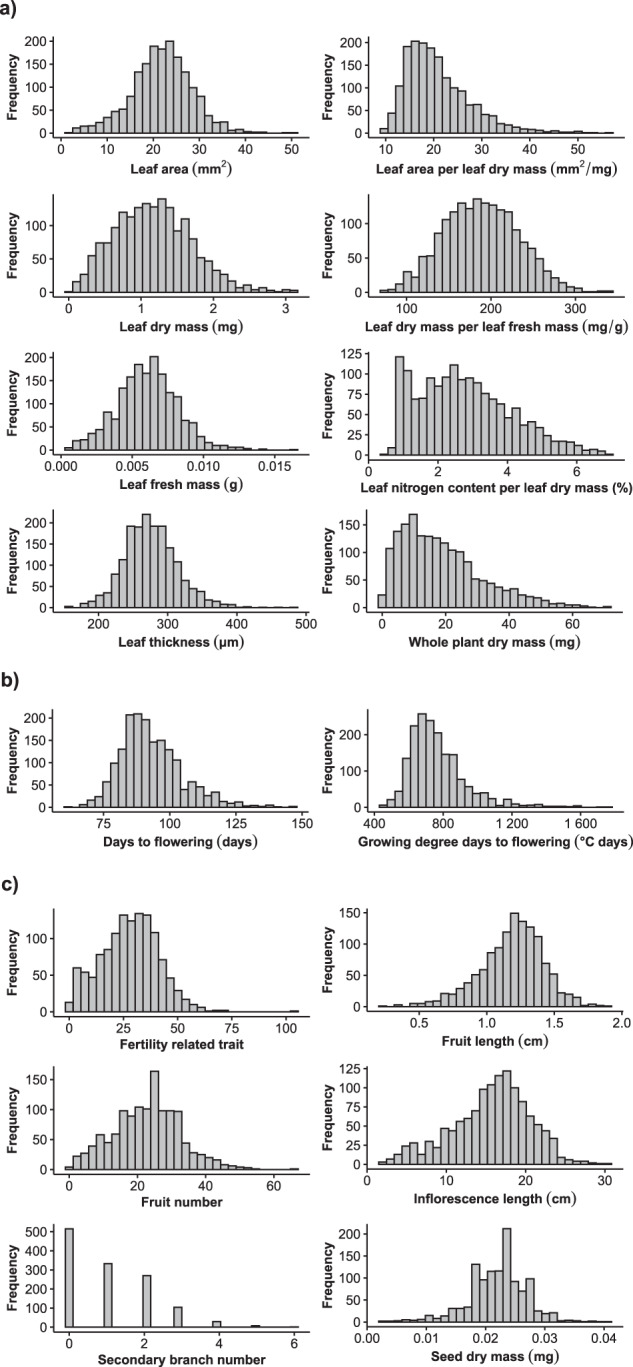
Histograms showing variation in vegetative (**a**) phenological (**b**) and reproductive (**c**) traits.

Functional trait measures followed standardized procedures described in Pérez-Harguindeguy *et al*.^23^, helping to reduce measurement variability. For dealing with measurement errors, we verified each trait for unrealistic extreme values by analyzing deviation from the mean. Values outside a range of µ ± 6σ (µ, mean; σ, standard deviation) were used to filter samples out (*N* = 2). Because outliers for reproductive traits were often associated with intense herbivory damage, we defined a qualitative herbivory index, ranging from zero to five, to each photographed plant according to the number of herbivores and their traces (*e.g*., damaged inflorescence stem and/or fruits) that could be detected in the images. Herbivory index for individual plants can be found in the phenotypic data record of the AraDiv dataset and may be used for filtering out possibly biased samples with high herbivory index (*i.e*., 35 samples with 4–5 herbivory index). The intraspecific phenotypic variation assessed in this study is shown in Fig. 4.

### NIRS prediction for LNC

NIRS prediction accuracy was verified by comparing observed and predicted LNC values using Vasseur’s *et al*.^14^ model (Fig. 3b). Prediction performances were estimated through the coefficient of determination (R²) and the root mean square error (RMSE, %).

## Supplementary information

Supplementary Table 1

## Data Availability

No custom code has been used during the generation and processing of this dataset.
