# Supplementary material for: AraDiv: a dataset of functional traits and leaf hyperspectral reflectance of Arabidopsis thaliana
Source: Sci Data. 2023 May 24;10:314. doi: 10.1038/s41597-023-02189-w (PMC10209097; doi:10.1038/s41597-023-02189-w)
Supplement: Supplementary file 1 — Supplementary Table 1 [file 41597_2023_2189_MOESM1_ESM.docx]

**Supplementary Table 1**. List of the accessions harvested at flowering and at fruit maturation during the experiment. Information about each accession, extracted from the 1001 Genomes Project dataset (<http://1001genomes.org/>), is provided.

|  | **1001 Genomes ID** | **Name** | **Country** | **Latitude** | **Longitude** | **Harvest at flowering** | **Harvest at fruit maturation** |
| --- | --- | --- | --- | --- | --- | --- | --- |
| **1** | 159 | MAR2-3 | FRA | 47.35 | 3.93333 | yes | yes |
| **2** | 350 | TOU-A1-88 | FRA | 46.6667 | 4.11667 | yes | yes |
| **3** | 351 | TOU-A1-89 | FRA | 46.6667 | 4.11667 | yes | yes |
| **4** | 403 | Zdarec3 | CZE | 49.3667 | 16.2667 | yes | yes |
| **5** | 424 | Draha2 | CZE | 49.4112 | 16.2815 | yes | yes |
| **6** | 430 | Gr-1 | AUT | 47 | 15.5 | yes | no |
| **7** | 628 | LI-OF-061 | USA | 40.7777 | -72.9069 | yes | yes |
| **8** | 630 | LI-OF-065 | USA | 40.7777 | -72.9069 | yes | yes |
| **9** | 763 | Kar-1 | KGZ | 42.3 | 74.3667 | yes | no |
| **10** | 765 | Sus-1 | KGZ | 42.1833 | 73.4 | yes | no |
| **11** | 766 | Dja-1 | KGZ | 42.5833 | 73.6333 | no | yes |
| **12** | 768 | Zal-1 | KGZ | 42.8 | 76.35 | yes | yes |
| **13** | 772 | Neo-6 | TJK | 37.35 | 72.4667 | yes | yes |
| **14** | 801 | KYC-33 | USA | 37.9169 | -84.4639 | yes | no |
| **15** | 870 | MIC-31 | USA | 41.8266 | -86.4366 | yes | no |
| **16** | 915 | LIN S-5 | USA | 41.8972 | -71.4378 | yes | no |
| **17** | 932 | CHA-41 | USA | 42.3634 | -71.1445 | yes | yes |
| **18** | 991 | Ale-Stenar-41-1 | SWE | 55.3833 | 14.05 | yes | yes |
| **19** | 992 | Ale-Stenar-44-4 | SWE | 55.3833 | 14.05 | yes | yes |
| **20** | 997 | Ale-Stenar-56-14 | SWE | 55.3833 | 14.05 | yes | no |
| **21** | 1002 | Ale-Stenar-64-24 | SWE | 55.3833 | 14.05 | yes | no |
| **22** | 1006 | Ale-Stenar-77-31 | SWE | 55.3833 | 14.05 | yes | no |
| **23** | 1061 | Brösarp-11-135 | SWE | 55.7167 | 14.1333 | yes | yes |
| **24** | 1062 | Brösarp-15-138 | SWE | 55.7167 | 14.1333 | yes | no |
| **25** | 1063 | Brösarp-21-140 | SWE | 55.7167 | 14.1333 | yes | yes |
| **26** | 1066 | Brösarp-34-145 | SWE | 55.7167 | 14.1333 | yes | yes |
| **27** | 1070 | Brösarp-45-153 | SWE | 55.7167 | 14.1333 | yes | yes |
| **28** | 1158 | Aledal-6-49 | SWE | 56.7 | 16.5167 | yes | no |
| **29** | 1166 | Aledal-14-73 | SWE | 56.7 | 16.5167 | yes | no |
| **30** | 1254 | Tos-82-387 | SWE | 59.4333 | 17.0167 | yes | no |
| **31** | 1257 | Tos-95-393 | SWE | 59.4333 | 17.0167 | yes | no |
| **32** | 1313 | Ängsö-59-422 | SWE | 59.5667 | 16.8667 | yes | no |
| **33** | 1317 | Ängsö-74-430 | SWE | 59.5667 | 16.8667 | yes | no |
| **34** | 1552 | Sku-30 | SWE | 63.0833 | 18.3667 | yes | yes |
| **35** | 1829 | Mdn-1 | USA | 42.051 | -86.509 | yes | yes |
| **36** | 1853 | MNF-Pot-21 | USA | 43.595 | -86.2657 | yes | yes |
| **37** | 1872 | MNF-Pot-75 | USA | 43.595 | -86.2657 | yes | yes |
| **38** | 1890 | MNF-Riv-21 | USA | 43.5139 | -86.1859 | yes | no |
| **39** | 1925 | MNF-Che-2 | USA | 43.5251 | -86.1843 | yes | yes |
| **40** | 1954 | MNF-Jac-12 | USA | 43.5187 | -86.1739 | yes | yes |
| **41** | 2016 | MNF-Pin-39 | USA | 43.5356 | -86.1788 | yes | yes |
| **42** | 2057 | Map-42 | USA | 42.166 | -86.412 | yes | no |
| **43** | 2171 | Paw-26 | USA | 42.148 | -86.431 | yes | yes |
| **44** | 2278 | SLSP-35 | USA | 43.665 | -86.496 | yes | yes |
| **45** | 2317 | Ste-40 | USA | 42.03 | -86.514 | yes | yes |
| **46** | 4779 | UKSW06-179 | UK | 50.4 | -4.9 | yes | yes |
| **47** | 4807 | UKSW06-207 | UK | 50.4 | -4.9 | yes | no |
| **48** | 4884 | UKSW06-285 | UK | 50.3 | -4.9 | yes | yes |
| **49** | 4958 | UKSW06-360 | UK | 50.5 | -4.5 | yes | yes |
| **50** | 5104 | UKSE06-252 | UK | 51.3 | 0.5 | yes | yes |
| **51** | 5151 | UKSE06-325 | UK | 52.2 | -1.7 | yes | no |
| **52** | 5165 | UKSE06-362 | UK | 51.3 | 0.4 | yes | yes |
| **53** | 5210 | UKSE06-432 | UK | 51.2 | 0.3 | yes | yes |
| **54** | 5279 | UKSE06-541 | UK | 51.3 | 1.1 | yes | no |
| **55** | 5349 | UKSE06-639 | UK | 51.1 | 0.4 | yes | yes |
| **56** | 5353 | UKNW06-003 | UK | 54.5 | -3 | yes | yes |
| **57** | 5486 | UKNW06-233 | UK | 54.6 | -3.3 | yes | yes |
| **58** | 5577 | UKNW06-403 | UK | 54.7 | -3.4 | yes | yes |
| **59** | 5644 | UKNW06-481 | UK | 54.4 | -2.9 | yes | yes |
| **60** | 5741 | For-2 | UK | 56.6 | -4.1 | yes | no |
| **61** | 5768 | UKID63 | UK | 54.1 | -1.5 | yes | yes |
| **62** | 5772 | Set-1 | UK | 54.1 | -2.3 | yes | yes |
| **63** | 5784 | Ty-1 | UK | 56.4 | -5.2 | yes | yes |
| **64** | 5800 | UKID96 | UK | 57.4 | -5.5 | yes | yes |
| **65** | 5811 | UKID107 | UK | 52.9 | -3.1 | yes | yes |
| **66** | 5830 | App1-12 | SWE | 56.3333 | 15.9667 | yes | yes |
| **67** | 5831 | App1-14 | SWE | 56.3333 | 15.9667 | yes | no |
| **68** | 5832 | App1-16 | SWE | 56.3333 | 15.9667 | yes | yes |
| **69** | 5836 | Boo2-3 | SWE | 55.86 | 13.51 | yes | yes |
| **70** | 5837 | Bor-1 | CZE | 49.4013 | 16.2326 | yes | no |
| **71** | 5856 | Dör-10 | SWE | 63.0167 | 17.4914 | yes | yes |
| **72** | 5860 | Dra-3 | SWE | 62.6814 | 18.0165 | yes | yes |
| **73** | 5865 | Dra1-4 | SWE | 55.76 | 14.12 | yes | yes |
| **74** | 5867 | Dra2-1 | SWE | 55.76 | 14.12 | yes | yes |
| **75** | 5893 | DraIV 1-11 | CZE | 49.4112 | 16.2815 | yes | yes |
| **76** | 5907 | DraIV 2-9 | CZE | 49.4112 | 16.2815 | yes | yes |
| **77** | 5950 | DraIV 5-12 | CZE | 49.4112 | 16.2815 | yes | yes |
| **78** | 5984 | DraIV 6-13 | CZE | 49.4112 | 16.2815 | yes | yes |
| **79** | 6008 | Duk | CZE | 49.1 | 16.2 | yes | no |
| **80** | 6009 | Eden-1 | SWE | 62.877 | 18.177 | yes | yes |
| **81** | 6010 | Eden-5 | SWE | 62.877 | 18.177 | yes | yes |
| **82** | 6011 | Eden-6 | SWE | 62.877 | 18.177 | yes | yes |
| **83** | 6012 | Eden-7 | SWE | 62.877 | 18.177 | yes | yes |
| **84** | 6013 | Eden-9 | SWE | 62.877 | 18.177 | yes | yes |
| **85** | 6016 | Eds-1 | SWE | 62.9 | 18.4 | yes | yes |
| **86** | 6017 | Eds-9 | SWE | 62.9 | 18.4 | yes | yes |
| **87** | 6019 | Fjä1-2 | SWE | 56.06 | 14.29 | yes | yes |
| **88** | 6020 | Fjä1-5 | SWE | 56.06 | 14.29 | yes | yes |
| **89** | 6023 | Fly2-1 | SWE | 55.7509 | 13.3712 | yes | yes |
| **90** | 6024 | Fly2-2 | SWE | 55.7509 | 13.3712 | yes | no |
| **91** | 6025 | Gro-3 | SWE | 62.6437 | 17.7339 | yes | yes |
| **92** | 6030 | Grön-5 | SWE | 62.806 | 18.1896 | yes | yes |
| **93** | 6034 | Hov1-7 | SWE | 56.1 | 13.74 | yes | no |
| **94** | 6035 | Hov1-10 | SWE | 56.1 | 13.74 | yes | yes |
| **95** | 6036 | Hov3-2 | SWE | 56.1 | 13.74 | yes | yes |
| **96** | 6038 | Hov3-5 | SWE | 56.1 | 13.74 | yes | yes |
| **97** | 6039 | Hovdala-2 | SWE | 56.1 | 13.74 | yes | yes |
| **98** | 6040 | Kni-1 | SWE | 55.66 | 13.4 | yes | yes |
| **99** | 6041 | Lis-3 | SWE | 56.0328 | 14.775 | yes | yes |
| **100** | 6042 | Lom1-1 | SWE | 56.09 | 13.9 | yes | no |
| **101** | 6043 | Löv-1 | SWE | 62.801 | 18.079 | yes | yes |
| **102** | 6069 | Nyl-7 | SWE | 62.9513 | 18.2763 | yes | no |
| **103** | 6070 | Omn-1 | SWE | 62.9308 | 18.3448 | yes | no |
| **104** | 6071 | Omn-5 | SWE | 62.9308 | 18.3448 | yes | yes |
| **105** | 6073 | ÖMö1-7 | SWE | 56.1481 | 15.8155 | yes | yes |
| **106** | 6074 | Ör-1 | SWE | 56.4573 | 16.1308 | yes | no |
| **107** | 6076 | Rev-2 | SWE | 55.6942 | 13.4504 | yes | yes |
| **108** | 6077 | Rev-3 | SWE | 55.6942 | 13.4504 | yes | yes |
| **109** | 6085 | Sparta-1 | SWE | 55.7097 | 13.2145 | yes | no |
| **110** | 6086 | Sr:3 | SWE | 58.9 | 11.2 | yes | yes |
| **111** | 6087 | Stu-2 | SWE | 56.4666 | 16.1284 | yes | yes |
| **112** | 6091 | T1010 | SWE | 55.6525 | 13.215 | yes | yes |
| **113** | 6092 | T1020 | SWE | 55.6514 | 13.2233 | yes | no |
| **114** | 6094 | T1040 | SWE | 55.6494 | 13.2147 | yes | no |
| **115** | 6095 | T1050 | SWE | 55.6486 | 13.2161 | yes | yes |
| **116** | 6097 | T1070 | SWE | 55.6481 | 13.2264 | yes | yes |
| **117** | 6099 | T1090 | SWE | 55.6575 | 13.2386 | yes | no |
| **118** | 6100 | T1110 | SWE | 55.6 | 13.2 | yes | yes |
| **119** | 6101 | T1120 | SWE | 55.6 | 13.2 | yes | yes |
| **120** | 6102 | T1130 | SWE | 55.6 | 13.2 | yes | no |
| **121** | 6104 | T1160 | SWE | 55.7 | 13.2 | yes | yes |
| **122** | 6105 | T450 | SWE | 55.7967 | 13.1211 | yes | yes |
| **123** | 6106 | T460 | SWE | 55.7931 | 13.1186 | yes | yes |
| **124** | 6107 | T470 | SWE | 55.7942 | 13.1222 | no | yes |
| **125** | 6109 | T510 | SWE | 55.7936 | 13.1233 | yes | yes |
| **126** | 6111 | T530 | SWE | 55.7989 | 13.1219 | yes | yes |
| **127** | 6112 | T540 | SWE | 55.7967 | 13.1044 | yes | no |
| **128** | 6115 | T580 | SWE | 55.8 | 13.1367 | yes | yes |
| **129** | 6118 | T610 | SWE | 55.7 | 13.2 | yes | yes |
| **130** | 6119 | T620 | SWE | 55.7 | 13.2 | yes | yes |
| **131** | 6122 | T670 | SWE | 55.8364 | 13.3075 | yes | yes |
| **132** | 6123 | T680 | SWE | 55.8369 | 13.3033 | yes | no |
| **133** | 6125 | T710 | SWE | 55.8403 | 13.3106 | yes | yes |
| **134** | 6126 | T720 | SWE | 55.8411 | 13.3047 | yes | yes |
| **135** | 6128 | T740 | SWE | 55.8397 | 13.2881 | yes | no |
| **136** | 6131 | T780 | SWE | 55.8369 | 13.3181 | yes | no |
| **137** | 6133 | T800 | SWE | 55.8364 | 13.2906 | yes | yes |
| **138** | 6134 | T810 | SWE | 55.8383 | 13.2906 | yes | no |
| **139** | 6136 | T840 | SWE | 55.9336 | 13.5519 | yes | yes |
| **140** | 6137 | T850 | SWE | 55.9419 | 13.5603 | yes | yes |
| **141** | 6138 | T860 | SWE | 55.9403 | 13.5511 | yes | yes |
| **142** | 6140 | T880 | SWE | 55.9392 | 13.5539 | yes | yes |
| **143** | 6141 | T890 | SWE | 55.9414 | 13.5542 | yes | no |
| **144** | 6142 | T900 | SWE | 55.9428 | 13.5558 | yes | no |
| **145** | 6145 | T930 | SWE | 55.9497 | 13.5533 | yes | no |
| **146** | 6148 | T960 | SWE | 55.9319 | 13.5508 | yes | no |
| **147** | 6149 | T970 | SWE | 55.9281 | 13.5481 | yes | yes |
| **148** | 6150 | T980 | SWE | 55.9261 | 13.5319 | yes | no |
| **149** | 6151 | T990 | SWE | 55.6528 | 13.2244 | yes | yes |
| **150** | 6169 | TÅD 01 | SWE | 62.8714 | 18.3447 | yes | yes |
| **151** | 6172 | TÅD 04 | SWE | 62.8717 | 18.3436 | yes | yes |
| **152** | 6173 | TÅD 05 | SWE | 62.8717 | 18.3419 | yes | yes |
| **153** | 6174 | TÅD 06 | SWE | 62.8719 | 18.3422 | yes | yes |
| **154** | 6177 | TÄL 03 | SWE | 62.6322 | 17.69 | yes | yes |
| **155** | 6180 | TÄL 07 | SWE | 62.6322 | 17.6906 | yes | no |
| **156** | 6184 | TBÖ 01 | SWE | 62.8892 | 18.4522 | yes | yes |
| **157** | 6188 | TDr-1 | SWE | 55.7683 | 14.1386 | yes | yes |
| **158** | 6189 | TDr-2 | SWE | 55.7686 | 14.1383 | yes | yes |
| **159** | 6192 | TDr-5 | SWE | 55.7692 | 14.1369 | yes | yes |
| **160** | 6193 | TDr-7 | SWE | 55.7694 | 14.1347 | yes | yes |
| **161** | 6194 | TDr-8 | SWE | 55.7706 | 14.1342 | yes | no |
| **162** | 6201 | TDr-16 | SWE | 55.7719 | 14.1211 | yes | yes |
| **163** | 6202 | TDr-17 | SWE | 55.7717 | 14.1206 | yes | yes |
| **164** | 6203 | TDr-18 | SWE | 55.7714 | 14.1208 | yes | yes |
| **165** | 6210 | TEDEN 03 | SWE | 62.8839 | 18.1836 | yes | no |
| **166** | 6216 | TFÄ 06 | SWE | 63.0167 | 18.3283 | yes | yes |
| **167** | 6217 | TFÄ 07 | SWE | 63.0169 | 18.3283 | yes | yes |
| **168** | 6218 | TFÄ 08 | SWE | 63.0172 | 18.3283 | yes | yes |
| **169** | 6220 | TGR 01 | SWE | 62.806 | 18.1896 | yes | yes |
| **170** | 6235 | TOM 01 | SWE | 62.9611 | 18.3589 | yes | no |
| **171** | 6237 | TOM 03 | SWE | 62.9619 | 18.35 | yes | no |
| **172** | 6242 | Tomegap-2 | SWE | 55.7 | 13.2 | yes | yes |
| **173** | 6244 | TRÄ 01 | SWE | 62.9169 | 18.4728 | yes | no |
| **174** | 6258 | TV-10 | SWE | 55.5796 | 14.3336 | yes | no |
| **175** | 6284 | TV-38 | SWE | 55.5796 | 14.3336 | no | yes |
| **176** | 6390 | UduI 3-36 | CZE | 49.2771 | 16.6314 | yes | yes |
| **177** | 6396 | UduI 4-9 | CZE | 49.2771 | 16.6314 | yes | no |
| **178** | 6413 | Ull3-4 | SWE | 56.06 | 13.97 | yes | yes |
| **179** | 6424 | ZdrI 1-23 | CZE | 49.3853 | 16.2544 | yes | yes |
| **180** | 6830 | Kz-13 | KAZ | 49.5 | 73.1 | yes | yes |
| **181** | 6897 | Ag-0 | FRA | 45 | 1.3 | yes | yes |
| **182** | 6898 | An-1 | BEL | 51.2167 | 4.4 | yes | yes |
| **183** | 6903 | Bor-4 | CZE | 49.4013 | 16.2326 | yes | no |
| **184** | 6904 | Br-0 | CZE | 49.2 | 16.6166 | yes | yes |
| **185** | 6907 | CIBC-17 | UK | 51.4083 | -0.6383 | yes | yes |
| **186** | 6909 | Col-0 | USA | 38.3 | -92.3 | yes | yes |
| **187** | 6911 | Cvi-0 | CPV | 15.1111 | -23.6167 | yes | no |
| **188** | 6913 | Eden-2 | SWE | 62.877 | 18.177 | yes | yes |
| **189** | 6915 | Ei-2 | GER | 50.3 | 6.3 | yes | no |
| **190** | 6917 | Fäb-2 | SWE | 63.0165 | 18.3174 | yes | yes |
| **191** | 6919 | Ga-0 | GER | 50.3 | 8 | yes | yes |
| **192** | 6922 | Gu-0 | GER | 50.3 | 8 | yes | no |
| **193** | 6923 | HR-10 | UK | 51.4083 | -0.6383 | yes | yes |
| **194** | 6929 | Kondara | TJK | 38.48 | 68.49 | yes | yes |
| **195** | 6931 | Kz-9 | KAZ | 49.5 | 73.1 | yes | no |
| **196** | 6938 | Ms-0 | RUS | 55.7522 | 37.6322 | yes | no |
| **197** | 6940 | Mz-0 | GER | 50.3 | 8.3 | yes | no |
| **198** | 6943 | NFA-10 | UK | 51.4083 | -0.6383 | yes | no |
| **199** | 6944 | NFA-8 | UK | 51.4083 | -0.6383 | yes | yes |
| **200** | 6945 | Nok-3 | NED | 52.24 | 4.45 | yes | yes |
| **201** | 6951 | Pu2-23 | CZE | 49.42 | 16.36 | yes | no |
| **202** | 6956 | Pu2-7 | CZE | 49.42 | 16.36 | yes | yes |
| **203** | 6957 | Pu2-8 | CZE | 49.42 | 16.36 | yes | yes |
| **204** | 6958 | Ra-0 | FRA | 46 | 3.3 | yes | yes |
| **205** | 6963 | Sorbo | TJK | 38.35 | 68.48 | yes | yes |
| **206** | 6966 | Sq-1 | UK | 51.4083 | -0.6383 | yes | yes |
| **207** | 6967 | Sq-8 | UK | 51.4083 | -0.6383 | yes | yes |
| **208** | 6970 | Ts-1 | ESP | 41.7194 | 2.93056 | yes | yes |
| **209** | 6971 | Ts-5 | ESP | 41.7194 | 2.93056 | yes | yes |
| **210** | 6973 | Ull2-3 | SWE | 56.0648 | 13.9707 | yes | yes |
| **211** | 6974 | Ull2-5 | SWE | 56.0648 | 13.9707 | yes | no |
| **212** | 6975 | Uod-1 | AUT | 48.3 | 14.45 | yes | yes |
| **213** | 6976 | Uod-7 | AUT | 48.3 | 14.45 | yes | yes |
| **214** | 6979 | Wei-0 | SUI | 47.25 | 8.26 | yes | yes |
| **215** | 6984 | Zdr-1 | CZE | 49.3853 | 16.2544 | yes | yes |
| **216** | 6986 | Abd-0 | UK | 57.1539 | -2.2207 | yes | yes |
| **217** | 6987 | Ak-1 | GER | 48.0683 | 7.62551 | yes | no |
| **218** | 6989 | Alst-1 | UK | 54.8 | -2.4333 | yes | yes |
| **219** | 6990 | Amel-1 | NED | 53.448 | 5.73 | yes | yes |
| **220** | 6992 | Ang-0 | BEL | 50.3 | 5.3 | yes | yes |
| **221** | 6997 | Appt-1 | NED | 51.8333 | 5.5833 | yes | yes |
| **222** | 7000 | Aa-0 | GER | 50.9167 | 9.57073 | yes | yes |
| **223** | 7002 | Baa-1 | NED | 51.3333 | 6.1 | yes | no |
| **224** | 7003 | Bs-1 | SUI | 47.5 | 7.5 | yes | no |
| **225** | 7008 | Benk-1 | NED | 52 | 5.675 | yes | yes |
| **226** | 7013 | Bd-0 | GER | 52.4584 | 13.287 | yes | no |
| **227** | 7014 | Ba-1 | UK | 56.5459 | -4.79821 | yes | yes |
| **228** | 7025 | Bl-1 | ITA | 44.5041 | 11.3396 | yes | yes |
| **229** | 7026 | Boot-1 | UK | 54.4 | -3.2667 | yes | yes |
| **230** | 7028 | Bch-1 | GER | 49.5166 | 9.3166 | yes | yes |
| **231** | 7031 | Bsch-0 | GER | 50.0167 | 8.6667 | yes | yes |
| **232** | 7036 | Bu-0 | GER | 50.5 | 9.5 | yes | no |
| **233** | 7062 | Ca-0 | GER | 50.2981 | 8.26607 | yes | no |
| **234** | 7071 | Chat-1 | FRA | 48.0717 | 1.33867 | yes | yes |
| **235** | 7072 | Chi-0 | RUS | 53.7502 | 34.7361 | yes | no |
| **236** | 7077 | Co-1 | POR | 40.12 | -8.25 | yes | yes |
| **237** | 7081 | Co | POR | 40.2077 | -8.42639 | yes | yes |
| **238** | 7092 | Com-1 | FRA | 49.416 | 2.823 | yes | yes |
| **239** | 7102 | Do-0 | GER | 50.7224 | 8.2372 | yes | no |
| **240** | 7106 | Dr-0 | GER | 51.051 | 13.7336 | yes | no |
| **241** | 7117 | El-0 | GER | 51.5105 | 9.68253 | yes | yes |
| **242** | 7119 | En-2 | GER | 50 | 8.5 | yes | yes |
| **243** | 7126 | Es-0 | FIN | 60.1997 | 24.5682 | yes | yes |
| **244** | 7127 | Est | EST | 58.6656 | 24.9871 | yes | no |
| **245** | 7130 | Et-0 | FRA | 44.6447 | 2.56481 | yes | yes |
| **246** | 7133 | Fr-2 | GER | 50.1102 | 8.6822 | yes | yes |
| **247** | 7143 | Gel-1 | NED | 51.0167 | 5.86667 | yes | no |
| **248** | 7147 | Gie-0 | GER | 50.584 | 8.67825 | yes | yes |
| **249** | 7158 | Gr-5 | AUT | 47 | 15.5 | yes | yes |
| **250** | 7162 | Hs-0 | GER | 52.24 | 9.44 | yes | yes |
| **251** | 7163 | Ha-0 | GER | 52.3721 | 9.73569 | yes | yes |
| **252** | 7169 | Hh-0 | GER | 54.4175 | 9.88682 | yes | yes |
| **253** | 7177 | Jm-0 | CZE | 49 | 15 | yes | yes |
| **254** | 7181 | Je-0 | GER | 50.927 | 11.587 | yes | yes |
| **255** | 7186 | Kn-0 | LTU | 54.8969 | 23.8924 | yes | yes |
| **256** | 7203 | Krot-0 | GER | 49.631 | 11.5722 | yes | no |
| **257** | 7207 | Kyoto | JPN | 35.0085 | 135.752 | yes | yes |
| **258** | 7208 | Lan-0 | UK | 55.6739 | -3.78181 | yes | yes |
| **259** | 7209 | La-0 | POL | 52.7333 | 15.2333 | yes | yes |
| **260** | 7217 | Lm-2 | FRA | 48 | 0.5 | yes | yes |
| **261** | 7218 | Le-0 | NED | 52.1611 | 4.49015 | yes | yes |
| **262** | 7223 | Li-2:1 | GER | 50.3833 | 8.0666 | yes | yes |
| **263** | 7231 | Li-7 | GER | 50.3833 | 8.0666 | no | yes |
| **264** | 7236 | Litva | LTU | NA | NA | yes | yes |
| **265** | 7244 | Mnz-0 | GER | 50.001 | 8.26664 | yes | no |
| **266** | 7250 | Me-0 | GER | 51.9183 | 10.1138 | yes | yes |
| **267** | 7255 | Mh-0 | POL | 50.95 | 20.5 | yes | yes |
| **268** | 7258 | Nw-0 | GER | 50.5 | 8.5 | yes | yes |
| **269** | 7268 | Np-0 | GER | 52.6969 | 10.981 | yes | yes |
| **270** | 7276 | Ob-0 | GER | 50.2 | 8.5833 | yes | no |
| **271** | 7280 | Old-1 | GER | 53.1667 | 8.2 | yes | no |
| **272** | 7282 | Or-0 | GER | 50.3827 | 8.01161 | yes | yes |
| **273** | 7287 | Ove-0 | GER | 53.3422 | 8.42255 | yes | yes |
| **274** | 7296 | Petergof | RUS | 59 | 29 | yes | no |
| **275** | 7298 | Pi-0 | AUT | 47.04 | 10.51 | yes | yes |
| **276** | 7305 | Pt-0 | GER | 53.476 | 10.6065 | yes | yes |
| **277** | 7306 | Pog-0 | CAN | 49.2655 | -123.206 | yes | yes |
| **278** | 7307 | Pn-0 | FRA | 48.0653 | -2.96591 | yes | yes |
| **279** | 7314 | Ragl-1 | UK | 54.3512 | -3.41697 | yes | no |
| **280** | 7316 | Rhen-1 | NED | 51.9667 | 5.56667 | yes | yes |
| **281** | 7319 | Rome-1 | ITA | 42 | 12.1 | yes | yes |
| **282** | 7323 | Rubezhnoe-1 | UKR | 49 | 38.28 | yes | no |
| **283** | 7327 | Sf-1 | ESP | 41.7833 | 3.03333 | yes | yes |
| **284** | 7328 | Sf-2 | ESP | 41.7833 | 3.03333 | yes | yes |
| **285** | 7333 | Sei-0 | ITA | 46.5438 | 11.5614 | yes | no |
| **286** | 7337 | Si-0 | GER | 50.8738 | 8.02341 | yes | yes |
| **287** | 7343 | Sp-0 | GER | 52.5339 | 13.181 | yes | yes |
| **288** | 7347 | Stw-0 | RUS | 52 | 36 | yes | no |
| **289** | 7353 | Tha-1 | NED | 52.08 | 4.3 | yes | yes |
| **290** | 7354 | Ting-1 | SWE | 56.5 | 14.9 | yes | no |
| **291** | 7356 | Tol-0 | USA | 41.6639 | -83.5553 | yes | yes |
| **292** | 7372 | Tscha-1 | AUT | 47.0748 | 9.9042 | yes | yes |
| **293** | 7373 | Tsu-0 | JPN | 34.43 | 136.31 | yes | no |
| **294** | 7378 | Uk-1 | GER | 48.0333 | 7.7667 | yes | yes |
| **295** | 7382 | Utrecht | NED | 52.0918 | 5.1145 | yes | no |
| **296** | 7383 | Van-0 | CAN | 49.2655 | -123.206 | yes | yes |
| **297** | 7384 | Ven-1 | NED | 52.0333 | 5.55 | yes | yes |
| **298** | 7387 | Vind-1 | UK | 54.9902 | -2.3671 | yes | yes |
| **299** | 7394 | Wa-1 | POL | 52.3 | 21 | yes | no |
| **300** | 7396 | Ws-0.2 | RUS | 52.3 | 30 | yes | no |
| **301** | 7404 | Wc-1 | GER | 52.6 | 10.0667 | yes | yes |
| **302** | 7411 | Wl-0 | GER | 47.9299 | 10.8134 | yes | yes |
| **303** | 7413 | Wil-2 | LTU | 54.6833 | 25.3167 | yes | no |
| **304** | 7416 | Yo-0 | USA | 37.45 | -119.35 | yes | yes |
| **305** | 7417 | Zu-0 | SUI | 47.3667 | 8.55 | yes | yes |
| **306** | 7418 | Zu-1 | SUI | 47.3667 | 8.55 | yes | yes |
| **307** | 7419 | Db-1 | GER | 50.3058 | 8.32213 | yes | no |
| **308** | 7424 | Jl-3 | CZE | 49.2 | 16.6166 | yes | yes |
| **309** | 7430 | Nc-1 | FRA | 48.6167 | 6.25 | yes | yes |
| **310** | 7461 | H55 | CZE | 49 | 15 | yes | no |
| **311** | 7477 | WAR | USA | 41.7302 | -71.2825 | yes | no |
| **312** | 7514 | RRS-7 | USA | 41.5609 | -86.4251 | yes | yes |
| **313** | 7516 | Vår2-1 | SWE | 55.58 | 14.334 | yes | yes |
| **314** | 7520 | Lp2-2 | CZE | 49.38 | 16.81 | yes | no |
| **315** | 7521 | Lp2-6 | CZE | 49.38 | 16.81 | yes | no |
| **316** | 7525 | Rmx-A180 | USA | 42.036 | -86.511 | yes | yes |
| **317** | 7717 | KNO1.37 | USA | 41.273 | -86.625 | yes | no |
| **318** | 8077 | PT2.21 | USA | 41.3423 | -86.7368 | yes | yes |
| **319** | 8132 | RMX3.22 | USA | 42.036 | -86.511 | yes | yes |
| **320** | 8171 | RMX4.118 | USA | 42.036 | -86.511 | yes | yes |
| **321** | 8214 | Gy-0 | FRA | 49 | 2 | yes | yes |
| **322** | 8222 | Lis-2 | SWE | 56.0328 | 14.775 | yes | no |
| **323** | 8227 | THÖ 03 | SWE | 62.7989 | 17.9103 | yes | yes |
| **324** | 8230 | Algutsrum | SWE | 56.68 | 16.5 | yes | yes |
| **325** | 8231 | Brö1-6 | SWE | 56.3 | 16 | yes | yes |
| **326** | 8234 | Gul1-2 | SWE | 56.4606 | 15.8127 | yes | yes |
| **327** | 8235 | Hod | CZE | 48.8 | 17.1 | yes | yes |
| **328** | 8236 | HSm | CZE | 49.33 | 15.76 | yes | yes |
| **329** | 8237 | Kävlinge-1 | SWE | 55.8 | 13.1 | yes | yes |
| **330** | 8240 | Kulturen-1 | SWE | 55.705 | 13.196 | yes | yes |
| **331** | 8241 | Liarum | SWE | 55.9473 | 13.821 | yes | yes |
| **332** | 8242 | Lillö-1 | SWE | 56.1494 | 15.7884 | yes | yes |
| **333** | 8243 | PHW-2 | ITA | 43.7703 | 11.2547 | yes | yes |
| **334** | 8244 | PHW-34 | FRA | 48.6103 | 2.3086 | yes | yes |
| **335** | 8246 | NC-6 | USA | 35 | -79.18 | yes | yes |
| **336** | 8247 | San-2 | SWE | 56.07 | 13.74 | yes | no |
| **337** | 8249 | Vimmerby | SWE | 57.7 | 15.8 | yes | yes |
| **338** | 8256 | Bå1-2 | SWE | 56.4 | 12.9 | yes | yes |
| **339** | 8264 | Bla-1 | ESP | 41.6833 | 2.8 | yes | yes |
| **340** | 8283 | Dra3-1 | SWE | 55.76 | 14.12 | yes | yes |
| **341** | 8285 | DraIII-1 | CZE | 49.4112 | 16.2815 | yes | yes |
| **342** | 8307 | Hovdala-6 | SWE | 56.1 | 13.74 | yes | yes |
| **343** | 8311 | In-0 | AUT | 47.5 | 11.5 | yes | no |
| **344** | 8312 | Is-0 | GER | 50.5 | 7.5 | yes | yes |
| **345** | 8326 | Lis-1 | SWE | 56.0328 | 14.775 | yes | no |
| **346** | 8334 | Lu-1 | SWE | 55.71 | 13.2 | yes | yes |
| **347** | 8335 | Lund | SWE | 55.71 | 13.2 | yes | no |
| **348** | 8337 | Mir-0 | ITA | 44 | 12.37 | yes | no |
| **349** | 8343 | Na-1 | FRA | 47.5 | 1.5 | yes | no |
| **350** | 8354 | Per-1 | RUS | 58 | 56.3167 | yes | no |
| **351** | 8357 | Pla-0 | ESP | 41.5 | 2.25 | yes | yes |
| **352** | 8365 | Rak-2 | CZE | 49 | 16 | yes | no |
| **353** | 8366 | Rd-0 | GER | 50.5 | 8.5 | yes | yes |
| **354** | 8369 | Rev-1 | SWE | 55.6942 | 13.4504 | yes | yes |
| **355** | 8376 | Sanna-2 | SWE | 62.69 | 18 | yes | yes |
| **356** | 8386 | Sr:5 | SWE | 58.9 | 11.2 | yes | yes |
| **357** | 8387 | St-0 | SWE | 59 | 18 | yes | no |
| **358** | 8419 | Wil-1 | LTU | 54.6833 | 25.3167 | yes | yes |
| **359** | 8420 | Kelsterbach-4 | GER | 50.0667 | 8.5333 | yes | yes |
| **360** | 8427 | Ull2-13 | SWE | 56.0648 | 13.9707 | yes | no |
| **361** | 9027 | RMX413.85 | USA | 42.036 | -86.511 | yes | no |
| **362** | 9057 | Vinslöv | SWE | 56.1 | 13.9167 | yes | yes |
| **363** | 9058 | Västervik | SWE | 57.75 | 16.6333 | yes | no |
| **364** | 9069 | Xan-5 | AZE | 38.6536 | 48.7992 | yes | no |
| **365** | 9070 | Xan-6 | AZE | 38.6536 | 48.7992 | yes | no |
| **366** | 9075 | Lerik1-4 | AZE | 38.7406 | 48.6131 | no | yes |
| **367** | 9078 | Lerik1-7 | AZE | 38.7406 | 48.6131 | yes | yes |
| **368** | 9079 | Lerik2-1 | AZE | 38.7833 | 48.5517 | yes | yes |
| **369** | 9081 | Lerik2-3 | AZE | 38.7833 | 48.5517 | yes | yes |
| **370** | 9089 | Nar-3 | AZE | 38.9522 | 48.925 | yes | yes |
| **371** | 9091 | Nar-5 | AZE | 38.9522 | 48.925 | yes | yes |
| **372** | 9095 | Istisu-5 | AZE | 38.9786 | 48.5594 | yes | no |
| **373** | 9099 | Istisu-9 | AZE | 38.9786 | 48.5594 | yes | yes |
| **374** | 9102 | Lag1-4 | GEO | 41.8296 | 46.2831 | yes | yes |
| **375** | 9103 | Lag1-5 | GEO | 41.8296 | 46.2831 | yes | yes |
| **376** | 9104 | Lag1-6 | GEO | 41.8296 | 46.2831 | yes | no |
| **377** | 9106 | Lag1-8 | GEO | 41.8296 | 46.2831 | yes | no |
| **378** | 9111 | Lag2-4 | GEO | 41.8296 | 46.2831 | yes | no |
| **379** | 9121 | Bak-5 | GEO | 41.7942 | 43.4767 | yes | yes |
| **380** | 9125 | Geg-14 | ARM | 40.1408 | 44.8203 | yes | no |
| **381** | 9298 | Edi-1 | UK | 55.9681 | -3.21833 | yes | yes |
| **382** | 9314 | Gol-2 | UK | 57.9672 | -3.96722 | yes | yes |
| **383** | 9321 | Ådal 1 | SWE | 62.8622 | 18.336 | yes | no |
| **384** | 9323 | Ådal 3 | SWE | 62.8622 | 18.336 | yes | yes |
| **385** | 9332 | Bar 1 | SWE | 62.8698 | 18.381 | yes | yes |
| **386** | 9336 | Bön 1 | SWE | 62.8794 | 18.4473 | yes | yes |
| **387** | 9339 | Böt 1 | SWE | 57.7133 | 15.0689 | yes | no |
| **388** | 9343 | Dju-1 | SWE | 57.3089 | 18.1512 | yes | yes |
| **389** | 9363 | EdJ 2 | SWE | 62.9147 | 18.4045 | yes | yes |
| **390** | 9369 | EkS 2 | SWE | 57.6781 | 14.9986 | yes | yes |
| **391** | 9370 | EkS 3 | SWE | 57.6781 | 14.9986 | yes | no |
| **392** | 9371 | FäL 1 | SWE | 63.016 | 18.3175 | yes | no |
| **393** | 9380 | FlyA 3 | SWE | 55.7488 | 13.3742 | yes | yes |
| **394** | 9381 | Fri 1 | SWE | 55.8106 | 14.2091 | yes | yes |
| **395** | 9382 | Fri 2 | SWE | 55.8106 | 14.2091 | yes | yes |
| **396** | 9383 | Fri 3 | SWE | 55.8106 | 14.2091 | yes | yes |
| **397** | 9386 | Grön 12 | SWE | 62.806 | 18.1896 | yes | no |
| **398** | 9394 | Hag-2 | SWE | 56.5804 | 16.4063 | yes | no |
| **399** | 9402 | Hel-3 | SWE | 57.8765 | 14.8549 | yes | yes |
| **400** | 9404 | HolA-1 1 | SWE | 55.7491 | 13.399 | yes | yes |
| **401** | 9405 | HolA-1 2 | SWE | 55.7491 | 13.399 | yes | yes |
| **402** | 9407 | HolA-2 2 | SWE | 55.7491 | 13.399 | no | yes |
| **403** | 9408 | Kal 1 | SWE | 56.047 | 13.9519 | yes | no |
| **404** | 9409 | Kia 1 | SWE | 56.0573 | 14.302 | yes | no |
| **405** | 9412 | Kor 3 | SWE | 57.2746 | 16.1494 | yes | no |
| **406** | 9413 | Kor 4 | SWE | 57.2746 | 16.1494 | yes | no |
| **407** | 9416 | Kru-3 | SWE | 57.7215 | 18.3837 | yes | yes |
| **408** | 9421 | Lan 1 | SWE | 55.9745 | 14.3997 | yes | no |
| **409** | 9427 | Näs 2 | SWE | 62.8815 | 18.4055 | yes | yes |
| **410** | 9433 | Nyl 13 | SWE | 62.9513 | 18.2763 | yes | no |
| **411** | 9437 | Puk-2 | SWE | 56.1633 | 14.6806 | yes | no |
| **412** | 9450 | Spro 1 | SWE | 57.2545 | 18.2109 | yes | yes |
| **413** | 9451 | Spro 2 | SWE | 57.2545 | 18.2109 | yes | no |
| **414** | 9452 | Spro 3 | SWE | 57.2545 | 18.2109 | yes | yes |
| **415** | 9454 | Stenk-3 | SWE | 57.8009 | 18.5162 | yes | no |
| **416** | 9455 | Stenk-4 | SWE | 57.8009 | 18.5162 | yes | yes |
| **417** | 9470 | Tur-4 | SWE | 57.6511 | 14.8043 | yes | no |
| **418** | 9471 | Ull-A-1 | SWE | 56.0648 | 13.9707 | yes | yes |
| **419** | 9481 | Yst-1 | SWE | 55.4242 | 13.8484 | yes | yes |
| **420** | 9503 | 11C1 | UK | 55.8877 | -3.21072 | yes | yes |
| **421** | 9506 | IP-Alo-0 | POR | 40.11 | -7.47 | yes | yes |
| **422** | 9507 | IP-Coa-0 | POR | 38.45 | -7.5 | yes | yes |
| **423** | 9508 | IP-Mos-1 | POR | 40.04 | -7.11 | yes | yes |
| **424** | 9509 | IP-Reg-0 | POR | 39.29 | -7.4 | yes | yes |
| **425** | 9510 | IP-Rei-0 | POR | 38.75 | -7.59 | yes | yes |
| **426** | 9511 | IP-Vav-0 | POR | 38.53 | -8.02 | yes | no |
| **427** | 9512 | IP-Vid-1 | POR | 38.22 | -7.84 | yes | no |
| **428** | 9513 | IP-Adc-5 | ESP | 38.77 | -4.07 | yes | yes |
| **429** | 9514 | IP-Adm-0 | ESP | 39.15 | -4.54 | yes | no |
| **430** | 9515 | IP-Ala-0 | ESP | 39.72 | -6.89 | yes | yes |
| **431** | 9517 | IP-All-0 | ESP | 42.19 | -7.8 | yes | yes |
| **432** | 9518 | IP-Alm-0 | ESP | 39.88 | -0.36 | yes | no |
| **433** | 9519 | IP-Ang-0 | ESP | 41.94 | 2.64 | yes | yes |
| **434** | 9520 | IP-Ara-4 | ESP | 41.7 | -3.68 | yes | no |
| **435** | 9521 | IP-Bar-1 | ESP | 41.43 | 2.13 | yes | yes |
| **436** | 9522 | IP-Bea-0 | ESP | 36.52 | -5.27 | yes | yes |
| **437** | 9523 | IP-Ben-0 | ESP | 38.37 | -2.66 | yes | yes |
| **438** | 9524 | IP-Ber-0 | ESP | 42.52 | -0.56 | yes | no |
| **439** | 9525 | IP-Bis-0 | ESP | 42.49 | 0.54 | yes | no |
| **440** | 9526 | IP-Cab-3 | ESP | 41.54 | 2.39 | yes | yes |
| **441** | 9527 | IP-Cad-0 | ESP | 40.37 | -5.74 | yes | yes |
| **442** | 9529 | IP-Cap-1 | ESP | 36.97 | -3.36 | yes | yes |
| **443** | 9530 | IP-Car-1 | ESP | 38.25 | -4.32 | yes | no |
| **444** | 9531 | IP-Cdc-3 | ESP | 41.21 | -4.54 | yes | yes |
| **445** | 9532 | IP-Cdo-0 | ESP | 42.23 | -4.64 | yes | yes |
| **446** | 9534 | IP-Cmo-3 | ESP | 40.05 | -4.65 | yes | yes |
| **447** | 9535 | IP-Coc-1 | ESP | 42.31 | 3.19 | yes | no |
| **448** | 9536 | IP-Cor-0 | ESP | 40.83 | -2 | yes | no |
| **449** | 9537 | IP-Cum-1 | ESP | 38.07 | -6.66 | yes | yes |
| **450** | 9539 | IP-Deh-1 | ESP | 40.29 | -6.67 | yes | yes |
| **451** | 9540 | IP-Elb-0 | ESP | 41.81 | 2.34 | yes | yes |
| **452** | 9541 | IP-Fue-2 | ESP | 38.26 | -5.42 | yes | yes |
| **453** | 9542 | IP-Fun-0 | ESP | 40.79 | -4.05 | yes | no |
| **454** | 9543 | IP-Gra-0 | ESP | 36.77 | -5.39 | yes | no |
| **455** | 9544 | IP-Gua-1 | ESP | 39.4 | -5.33 | yes | yes |
| **456** | 9545 | IP-Her-12 | ESP | 39.4 | -5.78 | yes | no |
| **457** | 9546 | IP-Hom-4 | ESP | 40.82 | -1.68 | yes | yes |
| **458** | 9547 | IP-Hor-0 | ESP | 41.67 | 2.62 | yes | no |
| **459** | 9548 | IP-Hoy-0 | ESP | 40.4 | -5 | yes | yes |
| **460** | 9549 | IP-Hum-2 | ESP | 42.23 | -3.69 | yes | no |
| **461** | 9551 | IP-Jim-1 | ESP | 42.28 | -5.92 | yes | no |
| **462** | 9553 | IP-Ldd-0 | ESP | 41.58 | -4.71 | yes | yes |
| **463** | 9554 | IP-Lso-0 | ESP | 38.86 | -3.16 | yes | no |
| **464** | 9556 | IP-Men-2 | ESP | 39.66 | -4.34 | yes | yes |
| **465** | 9557 | IP-Moa-0 | ESP | 42.46 | 0.7 | yes | no |
| **466** | 9558 | IP-Moc-11 | ESP | 41.57 | -5.64 | yes | yes |
| **467** | 9559 | IP-Mon-5 | ESP | 38.06 | -4.38 | yes | yes |
| **468** | 9560 | IP-Mot-0 | ESP | 38.19 | -6.24 | yes | yes |
| **469** | 9561 | IP-Mun-0 | ESP | 40.71 | -5.04 | yes | yes |
| **470** | 9562 | IP-Mur-0 | ESP | 41.67 | 2 | yes | yes |
| **471** | 9564 | IP-Nog-17 | ESP | 40.45 | -1.6 | yes | no |
| **472** | 9565 | IP-Orb-10 | ESP | 42.97 | -1.23 | yes | yes |
| **473** | 9567 | IP-Pal-0 | ESP | 42.34 | 1.3 | yes | yes |
| **474** | 9568 | IP-Pan-0 | ESP | 42.76 | -0.23 | yes | no |
| **475** | 9569 | IP-Pds-1 | ESP | 42.87 | -6.45 | yes | yes |
| **476** | 9571 | IP-Pro-0 | ESP | 43.28 | -6.01 | yes | yes |
| **477** | 9573 | IP-Rds-0 | ESP | 41.86 | 2.99 | yes | no |
| **478** | 9574 | IP-Rel-0 | ESP | 38.6 | -2.7 | yes | yes |
| **479** | 9576 | IP-Rev-0 | ESP | 40.86 | -4.11 | yes | no |
| **480** | 9577 | IP-Ria-0 | ESP | 42.34 | 2.17 | yes | no |
| **481** | 9578 | IP-Sac-0 | ESP | 42.13 | -6.7 | yes | yes |
| **482** | 9579 | IP-San-10 | ESP | 38.33 | -3.51 | yes | yes |
| **483** | 9581 | IP-Sdv-3 | ESP | 42.84 | -5.12 | yes | yes |
| **484** | 9582 | IP-Ses-0 | ESP | 41.48 | -1.63 | yes | no |
| **485** | 9583 | IP-Sne-0 | ESP | 37.09 | -3.38 | yes | yes |
| **486** | 9584 | IP-Stp-0 | ESP | 41.19 | -3.58 | yes | yes |
| **487** | 9585 | IP-Svi-0 | ESP | 43.4 | -7.39 | yes | no |
| **488** | 9586 | IP-Tam-0 | ESP | 41.03 | -3.27 | yes | no |
| **489** | 9587 | IP-Tdc-0 | ESP | 41.5 | -1.88 | yes | no |
| **490** | 9588 | IP-Tol-7 | ESP | 42.11 | 0.6 | yes | no |
| **491** | 9589 | IP-Tor-1 | ESP | 41.6 | -2.83 | yes | yes |
| **492** | 9590 | IP-Trs-0 | ESP | 43.37 | -5.49 | yes | yes |
| **493** | 9591 | IP-Vad-0 | ESP | 42.86 | -3.59 | yes | yes |
| **494** | 9593 | IP-Vaz-0 | ESP | 42.26 | -2.99 | yes | no |
| **495** | 9594 | IP-Vdm-0 | ESP | 42.04 | 1.01 | yes | yes |
| **496** | 9595 | IP-Vdt-0 | ESP | 40.89 | -5.5 | yes | yes |
| **497** | 9596 | IP-Ver-5 | ESP | 41.95 | -7.45 | yes | yes |
| **498** | 9597 | IP-Vig-1 | ESP | 42.31 | -2.53 | yes | no |
| **499** | 9598 | IP-Vim-0 | ESP | 41.88 | -6.51 | yes | no |
| **500** | 9599 | IP-Vin-0 | ESP | 42.8 | -5.77 | yes | yes |
| **501** | 9601 | IP-Voz-0 | ESP | 41.85 | -1.88 | yes | yes |
| **502** | 9602 | IP-Vpa-1 | ESP | 40.5 | -3.96 | yes | yes |
| **503** | 9606 | Aitba-1 | MAR | 31.48 | -7.45 | yes | yes |
| **504** | 9609 | Adam-1 | RUS | 51.41 | 59.98 | yes | yes |
| **505** | 9611 | Lesno-1 | RUS | 53.04 | 51.9 | yes | yes |
| **506** | 9612 | Lesno-2 | RUS | 53.04 | 51.94 | yes | yes |
| **507** | 9615 | Parti-1 | RUS | 52.99 | 52.16 | yes | yes |
| **508** | 9616 | Krazo-1 | RUS | 53.06 | 51.96 | yes | yes |
| **509** | 9617 | Karag-1 | RUS | 51.37 | 59.44 | yes | no |
| **510** | 9619 | Basta-1 | RUS | 51.84 | 79.48 | yes | yes |
| **511** | 9622 | Bijisk-4 | RUS | 52.52 | 85.27 | yes | yes |
| **512** | 9625 | Kolyv-2 | RUS | 51.31 | 82.59 | yes | yes |
| **513** | 9626 | Kolyv-3 | RUS | 51.36 | 82.59 | yes | yes |
| **514** | 9629 | K-oze-1 | RUS | 51.35 | 82.18 | yes | yes |
| **515** | 9630 | K-oze-3 | RUS | 51.34 | 82.16 | yes | yes |
| **516** | 9631 | Lebja-1 | RUS | 51.65 | 80.79 | yes | yes |
| **517** | 9632 | Lebja-2 | RUS | 51.67 | 80.82 | yes | yes |
| **518** | 9634 | Masl-1 | RUS | 54.13 | 81.31 | yes | yes |
| **519** | 9635 | Nosov-1 | RUS | 51.87 | 80.6 | yes | yes |
| **520** | 9636 | Noveg-1 | RUS | 51.75 | 80.82 | yes | no |
| **521** | 9637 | Noveg-2 | RUS | 51.77 | 80.85 | yes | no |
| **522** | 9638 | Noveg-3 | RUS | 51.73 | 80.86 | yes | yes |
| **523** | 9641 | Rakit-2 | RUS | 51.9 | 80.06 | yes | yes |
| **524** | 9643 | Sever-1 | RUS | 52.1 | 79.31 | yes | yes |
| **525** | 9644 | Zupan-1 | CRO | 45.07 | 18.72 | yes | yes |
| **526** | 9645 | Gradi-1 | CRO | 45.17 | 18.7 | yes | yes |
| **527** | 9647 | Basen-1 | ITA | 40.37 | 16.77 | yes | yes |
| **528** | 9649 | Bivio-1 | ITA | 39.13 | 16.17 | yes | yes |
| **529** | 9651 | Filet-1 | ITA | 40.68 | 14.87 | yes | no |
| **530** | 9653 | Giffo-1 | ITA | 38.44 | 16.13 | yes | yes |
| **531** | 9656 | Marti-1 | ITA | 40.64 | 17.31 | yes | yes |
| **532** | 9657 | Melic-1 | ITA | 38.45 | 16.04 | yes | yes |
| **533** | 9659 | Pigna-1 | ITA | 41.18 | 14.18 | yes | yes |
| **534** | 9660 | Sarno-1 | ITA | 40.84 | 14.57 | yes | yes |
| **535** | 9663 | Teano-1 | ITA | 41.33 | 14.09 | yes | yes |
| **536** | 9664 | Mitterberg-1-179 | ITA | 46.366 | 11.2832 | yes | yes |
| **537** | 9665 | Mitterberg-1-180 | ITA | 46.366 | 11.2832 | yes | yes |
| **538** | 9669 | Mitterberg-2-185 | ITA | 46.3668 | 11.2837 | yes | no |
| **539** | 9672 | Mitterberg-3-188 | ITA | 46.3668 | 11.2837 | yes | yes |
| **540** | 9678 | Mitterberg-4-194 | ITA | 46.3718 | 11.2866 | yes | no |
| **541** | 9686 | Castelfed-2-202 | ITA | 46.3378 | 11.2928 | yes | yes |
| **542** | 9697 | Dolen-1 | BUL | 41.62 | 23.94 | yes | yes |
| **543** | 9698 | Goced-1 | BUL | 41.57 | 23.85 | yes | yes |
| **544** | 9699 | Kolar-1 | BUL | 41.37 | 23.14 | yes | yes |
| **545** | 9701 | Ivano-1 | BUL | 43.7 | 25.91 | yes | yes |
| **546** | 9703 | Melni-1 | BUL | 41.53 | 23.39 | yes | yes |
| **547** | 9704 | Melni-2 | BUL | 41.53 | 23.39 | yes | yes |
| **548** | 9705 | Choto-1 | BUL | 41.5 | 23.33 | yes | yes |
| **549** | 9706 | Dospa-1 | BUL | 41.64 | 24.18 | yes | yes |
| **550** | 9707 | Podvi-1 | BUL | 41.57 | 24.84 | yes | yes |
| **551** | 9708 | Kardz-1 | BUL | 41.62 | 25.35 | no | yes |
| **552** | 9709 | Zerev-1-34 | BUL | 41.85 | 23.13 | yes | yes |
| **553** | 9710 | Zerev-1-35 | BUL | 41.85 | 23.13 | yes | yes |
| **554** | 9711 | Dolna-1-39 | BUL | 42.32 | 23.1 | yes | yes |
| **555** | 9713 | Stara-1 | BUL | 42.49 | 25.61 | yes | no |
| **556** | 9714 | Grivo-1 | BUL | 41.84 | 25.75 | yes | yes |
| **557** | 9716 | Leska-1-44 | BUL | 41.54 | 24.98 | yes | yes |
| **558** | 9717 | Kardz-2 | BUL | 41.66 | 25.47 | yes | yes |
| **559** | 9718 | Smolj-1 | BUL | 41.55 | 24.75 | yes | yes |
| **560** | 9719 | Koren-1 | BUL | 41.83 | 25.69 | yes | yes |
| **561** | 9720 | Malak-1 | BUL | 41.77 | 25.68 | yes | yes |
| **562** | 9722 | Groch-1 | BUL | 41.71 | 24.41 | yes | no |
| **563** | 9723 | Slavi-2 | BUL | 41.42 | 23.67 | yes | yes |
| **564** | 9725 | Epidauros-1 | GRC | 37.6 | 23.08 | yes | yes |
| **565** | 9726 | Faneronemi-3 | GRC | 37.07 | 22.04 | yes | yes |
| **566** | 9729 | Stiav-2 | SVK | 48.46 | 18.9 | yes | yes |
| **567** | 9730 | Bela-1 | SVK | 48.47 | 18.94 | yes | no |
| **568** | 9731 | Stiav-3 | SVK | 48.46 | 18.9 | yes | no |
| **569** | 9732 | Halca-1 | SVK | 48.47 | 18.96 | yes | yes |
| **570** | 9733 | Bela-2 | SVK | 48.47 | 18.94 | yes | no |
| **571** | 9735 | Bela-4 | SVK | 48.47 | 18.94 | yes | no |
| **572** | 9736 | Teiu-2 | ROU | 44.69 | 25.17 | yes | yes |
| **573** | 9737 | Ulies-1 | ROU | 45.95 | 22.62 | yes | no |
| **574** | 9738 | Bran-1 | ROU | 45.57 | 25.42 | yes | yes |
| **575** | 9741 | Orast-1 | ROU | 45.84 | 23.16 | yes | yes |
| **576** | 9743 | Furni-1 | ROU | 45.14 | 25 | yes | yes |
| **577** | 9744 | Iasi-1 | ROU | 47.16 | 27.59 | yes | yes |
| **578** | 9747 | Zabar-1 | SRB | 44.38 | 21.22 | yes | yes |
| **579** | 9748 | Zagub-1 | SRB | 44.23 | 21.71 | yes | yes |
| **580** | 9749 | Knjas-1 | SRB | 43.54 | 22.29 | yes | yes |
| **581** | 9754 | Sredn-1 | SRB | 44.66 | 21.37 | yes | yes |
| **582** | 9755 | Vajug-1 | SRB | 44.56 | 22.56 | yes | yes |
| **583** | 9756 | Staro-2 | SRB | 44.3 | 21.08 | yes | yes |
| **584** | 9757 | Staro-1 | SRB | 44.3 | 21.08 | yes | yes |
| **585** | 9758 | Altai-5 | CHN | 47.75 | 88.4 | yes | no |
| **586** | 9759 | Anz-0 | IRN | 37.47 | 49.47 | yes | yes |
| **587** | 9761 | Bik-1 | LBN | 33.92 | 35.7 | yes | yes |
| **588** | 9762 | Etna-2 | ITA | 37.69 | 14.98 | yes | no |
| **589** | 9764 | Qar-8a | LBN | 34.1 | 35.84 | yes | no |
| **590** | 9766 | Westkar-4 | KGZ | 42.26 | 74.16 | yes | no |
| **591** | 9768 | Ru4-16 | GER | 48.57 | 9.16 | yes | yes |
| **592** | 9769 | HE-1 | GER | 48.55 | 8.99 | yes | no |
| **593** | 9770 | KBG2-13 | GER | 48.53 | 9.01 | yes | yes |
| **594** | 9771 | Pfn-N2.2-6 | GER | 48.56 | 9.11 | yes | yes |
| **595** | 9772 | Hof-1 | GER | 48.41 | 8.85 | yes | yes |
| **596** | 9774 | Alt-1 | GER | 48.59 | 9.22 | yes | no |
| **597** | 9775 | Berg-1 | GER | 48.41 | 8.79 | yes | yes |
| **598** | 9776 | Fell3-7 | GER | 48.43 | 8.79 | yes | yes |
| **599** | 9777 | Gn-1 | GER | 48.57 | 9.17 | yes | yes |
| **600** | 9778 | Bach-7 | GER | 48.41 | 8.84 | yes | yes |
| **601** | 9779 | Bai-10 | GER | 48.5 | 8.78 | yes | no |
| **602** | 9780 | Fell2-4 | GER | 48.43 | 8.79 | yes | yes |
| **603** | 9781 | Kus2-2 | GER | 48.52 | 9.11 | yes | yes |
| **604** | 9782 | Lu3-30 | GER | 48.53 | 9.09 | yes | yes |
| **605** | 9784 | Erg2-6 | GER | 48.5 | 8.8 | yes | no |
| **606** | 9785 | Ha-HBT1-2 | GER | 48.54 | 9.02 | yes | yes |
| **607** | 9786 | Ha-P-13 | GER | 48.54 | 9.01 | yes | no |
| **608** | 9787 | HI-4 | GER | 48.5 | 9 | yes | yes |
| **609** | 9788 | KBG1-14 | GER | 48.53 | 9.01 | yes | no |
| **610** | 9789 | Obh-13 | GER | 48.39 | 8.96 | yes | no |
| **611** | 9790 | Gn2-3 | GER | 48.58 | 9.18 | yes | yes |
| **612** | 9791 | Haes-1 | GER | 48.6 | 9.2 | yes | yes |
| **613** | 9792 | Lu4-2 | GER | 48.54 | 9.09 | yes | no |
| **614** | 9793 | Ru-N2 | GER | 48.57 | 9.16 | yes | yes |
| **615** | 9794 | Tu-B1-2 | GER | 48.52 | 9.08 | yes | yes |
| **616** | 9795 | Wank-2 | GER | 48.5 | 9.11 | yes | yes |
| **617** | 9796 | Bach2-1 | GER | 48.41 | 8.84 | yes | yes |
| **618** | 9797 | Ha-HBT2-10 | GER | 48.54 | 9.02 | yes | no |
| **619** | 9798 | Ha-P2-1 | GER | 48.54 | 9.01 | yes | yes |
| **620** | 9799 | Hart-2 | GER | 48.39 | 8.85 | yes | yes |
| **621** | 9800 | Ha-S-B | GER | 48.54 | 9.01 | yes | yes |
| **622** | 9801 | Ha-SP-2 | GER | 48.54 | 9.01 | yes | no |
| **623** | 9802 | Kus3-1 | GER | 48.51 | 9.11 | yes | yes |
| **624** | 9803 | Muh-2 | GER | 48.42 | 8.76 | yes | yes |
| **625** | 9804 | Obe1-15 | GER | 48.45 | 8.87 | yes | yes |
| **626** | 9805 | Pfn-10 | GER | 48.54 | 9.09 | yes | yes |
| **627** | 9806 | Ru-2 | GER | 48.56 | 9.16 | yes | no |
| **628** | 9807 | Schl-7 | GER | 48.6 | 9.22 | yes | no |
| **629** | 9808 | Tu-B2-3 | GER | 48.52 | 9.08 | yes | no |
| **630** | 9810 | Tu-KS-7 | GER | 48.53 | 9.07 | yes | yes |
| **631** | 9811 | Tu-NK-12 | GER | 48.52 | 9.05 | yes | yes |
| **632** | 9812 | Tu-W1 | GER | 48.52 | 9.03 | yes | yes |
| **633** | 9813 | BI-4 | GER | 48.4 | 8.77 | yes | yes |
| **634** | 9814 | Fell1-10 | GER | 48.42 | 8.79 | yes | yes |
| **635** | 9815 | Ha-HBT3-11 | GER | 48.54 | 9.02 | yes | yes |
| **636** | 9817 | IP-Ace-0 | ESP | 39.84 | -6.6 | yes | yes |
| **637** | 9819 | IP-Amu-0 | ESP | 42.35 | -3.03 | yes | yes |
| **638** | 9820 | IP-Are-0 | ESP | 41 | -4.71 | yes | yes |
| **639** | 9821 | IP-Aru-0 | ESP | 41.81 | 2.49 | yes | yes |
| **640** | 9822 | IP-Aul-0 | ESP | 40.52 | -4.02 | yes | yes |
| **641** | 9823 | IP-Bae-0 | ESP | 43.34 | -5.84 | yes | yes |
| **642** | 9824 | IP-Bes-5 | ESP | 42.91 | -4.91 | yes | no |
| **643** | 9825 | IP-Boa-0 | ESP | 40.4 | -3.88 | yes | yes |
| **644** | 9826 | IP-Bor-0 | ESP | 42.49 | -6.71 | yes | yes |
| **645** | 9827 | IP-Bos-0 | ESP | 42.78 | 0.69 | yes | yes |
| **646** | 9830 | IP-Bus-0 | ESP | 36.97 | -3.28 | yes | yes |
| **647** | 9831 | IP-Cas-0 | ESP | 38.54 | -3.39 | yes | yes |
| **648** | 9832 | IP-Cat-0 | ESP | 40.54 | -3.69 | yes | no |
| **649** | 9833 | IP-Cha-0 | ESP | 40.38 | -4.21 | yes | yes |
| **650** | 9834 | IP-Cho-0 | ESP | 40.51 | -3.9 | yes | yes |
| **651** | 9835 | IP-Cir-0 | ESP | 40.61 | -6.57 | yes | yes |
| **652** | 9836 | IP-Cod-0 | ESP | 41.25 | -1.32 | yes | yes |
| **653** | 9837 | IP-Con-0 | ESP | 37.94 | -5.6 | yes | yes |
| **654** | 9838 | IP-Cot-0 | ESP | 41.83 | -5.38 | yes | yes |
| **655** | 9839 | IP-Coy-0 | ESP | 40.44 | -4.27 | yes | yes |
| **656** | 9840 | IP-Dar-0 | ESP | 41.13 | -1.43 | yes | yes |
| **657** | 9841 | IP-Ees-0 | ESP | 40.59 | -4.15 | yes | yes |
| **658** | 9843 | IP-Elp-0 | ESP | 40.53 | -3.92 | yes | yes |
| **659** | 9845 | IP-Evs-0 | ESP | 40.48 | -3.96 | yes | yes |
| **660** | 9846 | IP-Ezc-2 | ESP | 42.31 | -3.02 | yes | no |
| **661** | 9847 | IP-Fel-2 | ESP | 43.31 | -5.7 | yes | yes |
| **662** | 9848 | IP-Glo-1 | ESP | 40.11 | -5.77 | yes | no |
| **663** | 9850 | IP-Hec-0 | ESP | 42.86 | -0.7 | yes | yes |
| **664** | 9851 | IP-Hue-3 | ESP | 42.96 | -6.1 | yes | yes |
| **665** | 9852 | IP-Ini-0 | ESP | 40.46 | -3.75 | yes | yes |
| **666** | 9853 | IP-Lac-0 | ESP | 43.33 | -5.91 | yes | yes |
| **667** | 9854 | IP-Laf-1 | ESP | 43.36 | -5.88 | yes | yes |
| **668** | 9855 | IP-Lam-0 | ESP | 40.57 | -3.89 | yes | no |
| **669** | 9866 | IP-Mdd-0 | ESP | 41.89 | -2.79 | yes | no |
| **670** | 9867 | IP-Mie-1 | ESP | 40.94 | -3.22 | yes | no |
| **671** | 9868 | IP-Moe-0 | ESP | 41.78 | 2.37 | yes | yes |
| **672** | 9869 | IP-Moj-0 | ESP | 36.76 | -5.28 | yes | yes |
| **673** | 9870 | IP-Moz-0 | ESP | 41.91 | 0.17 | yes | yes |
| **674** | 9871 | IP-Nac-0 | ESP | 40.75 | -3.99 | yes | no |
| **675** | 9873 | IP-Ndc-0 | ESP | 37.94 | -5.45 | yes | yes |
| **676** | 9874 | IP-Oja-0 | ESP | 42.34 | -3 | yes | yes |
| **677** | 9876 | IP-Pad-0 | ESP | 41.34 | 0.99 | yes | no |
| **678** | 9877 | IP-Pdl-0 | ESP | 43.02 | -5.6 | yes | no |
| **679** | 9878 | IP-Pee-0 | ESP | 40.78 | -3.62 | yes | no |
| **680** | 9879 | IP-Per-0 | ESP | 37.6 | -1.12 | yes | no |
| **681** | 9880 | IP-Pib-1 | ESP | 42.72 | -3.44 | yes | no |
| **682** | 9881 | IP-Pie-0 | ESP | 40.46 | -5.32 | yes | yes |
| **683** | 9882 | IP-Pil-0 | ESP | 40.46 | -4.26 | yes | yes |
| **684** | 9883 | IP-Piq-0 | ESP | 42.1 | -2.56 | yes | yes |
| **685** | 9885 | IP-Prd-0 | ESP | 41.14 | -3.68 | yes | yes |
| **686** | 9886 | IP-Pru-0 | ESP | 42.38 | 1.73 | yes | no |
| **687** | 9887 | IP-Pun-0 | ESP | 40.4 | -4.77 | yes | no |
| **688** | 9888 | IP-Pva-1 | ESP | 40.93 | -3.31 | yes | yes |
| **689** | 9890 | IP-Rib-1 | ESP | 43.16 | -5.07 | yes | yes |
| **690** | 9891 | IP-Sal-0 | ESP | 41.93 | 2.92 | yes | yes |
| **691** | 9892 | IP-Sam-0 | ESP | 42.68 | -6.96 | yes | yes |
| **692** | 9894 | IP-Sen-0 | ESP | 42.59 | 0.76 | yes | yes |
| **693** | 9895 | IP-Sfb-6 | ESP | 41.78 | 2.57 | yes | yes |
| **694** | 9897 | IP-Smt-1 | ESP | 40.95 | -5.63 | yes | yes |
| **695** | 9898 | IP-Som-0 | ESP | 41.14 | -3.58 | yes | no |
| **696** | 9899 | IP-Tau-0 | ESP | 42.54 | 0.84 | yes | no |
| **697** | 9900 | IP-Tri-0 | ESP | 37.38 | -6.01 | yes | yes |
| **698** | 9901 | IP-Urd-1 | ESP | 42.27 | -2.98 | yes | yes |
| **699** | 9902 | IP-Usa-0 | ESP | 40.71 | -3.24 | yes | yes |
| **700** | 9903 | IP-Val-0 | ESP | 42.31 | -3.1 | yes | yes |
| **701** | 9904 | IP-Vas-0 | ESP | 40.95 | -3.31 | yes | yes |
| **702** | 9905 | IP-Ven-0 | ESP | 40.76 | -4.01 | yes | no |
| **703** | 9906 | IP-Mah-6 | ESP | 40 | 4.25 | yes | yes |
| **704** | 9910 | BRI-2 | FRA | 50.68 | 3.52 | yes | yes |
| **705** | 9918 | SAUL-24 | FRA | 47.43 | 5.21 | yes | yes |
| **706** | 9925 | RUM-20 | FRA | 48.91 | 4.52 | yes | no |
| **707** | 9928 | BEZ-9 | FRA | 44.12 | 3.77 | yes | no |
| **708** | 9933 | VED-10 | FRA | 43.74 | 3.89 | yes | yes |
| **709** | 9935 | BAU-15 | FRA | 50.6 | 2.93 | yes | yes |
| **710** | 9937 | CATS-6 | FRA | 50.79 | 2.69 | yes | yes |
| **711** | 9938 | WAV-8 | FRA | 50.65 | 2.99 | yes | no |
| **712** | 9943 | Cdm-0 | ESP | 39.73 | -5.74 | yes | yes |
| **713** | 9947 | Ped-0 | ESP | 40.74 | -3.9 | no | yes |
| **714** | 9959 | Shigu-2 | RUS | 53.33 | 49.48 | yes | yes |
| **715** | 9960 | Kidr-1 | RUS | 51.31 | 57.56 | yes | yes |
| **716** | 9966 | Monte-1 | ITA | 40.28 | 15.65 | yes | yes |
| **717** | 9968 | Timpo-1 | ITA | 39.27 | 16.27 | yes | yes |
| **718** | 9978 | Vezzano-2.2 | ITA | 46.6297 | 10.817 | yes | no |
| **719** | 9997 | Rue3-1-31 | GER | 48.56 | 9.16 | yes | yes |
| **720** | 10002 | TueWa1-2 | GER | 48.53 | 9.04 | yes | yes |
| **721** | 10020 | Jl-2 | CZE | 49.17 | 16.5 | yes | no |
